# Preparation and characterization of TiO_2_ based on wood templates

**DOI:** 10.1038/s41598-020-69440-x

**Published:** 2020-07-24

**Authors:** Yu Liu, Xiaodong Zhu, Diliang Yuan, Weicong Wang, Lijiao Gao

**Affiliations:** 1 College of Materials and Engineering, Northeast Forestry University, Harbin, 150040 Heilongjiang China; 2 Key Laboratory of Bio-Based Material Science and Technology (Ministry of Education), Northeast Forestry University, Harbin, 150040 Heilongjiang China

**Keywords:** Composites, Characterization and analytical techniques

## Abstract

Titanium dioxide (TiO_2_) was prepared from four natural wood spices that served as templates. The wood templates were impregnated by a titanium dioxide precursor and then underwent high-temperature calcination to obtain TiO_2_ with a wood-like hierarchical porous structure. The microstructure of TiO_2_ based on the wood template was characterized by scanning electron microscopy, X-ray diffraction and nitrogen adsorption–desorption tests. The formaldehyde adsorption and degradation properties of TiO_2_ based on a wood template are discussed. The results showed that TiO_2_ based on a wood template could effectively replicate the micro- and mesoscopic pore structure of wood, and the pore size distribution in the TiO_2_ ranged from 1 to 100 nm. The TiO_2_ that was prepared based on a wood template showed a certain adsorption effect on formaldehyde under visible light, and the photocatalytic degradation of a formaldehyde solution was achieved when irritated by ultraviolet light. In addition, the properties of the TiO_2_ prepared by different tree species was also different. The TiO_2_ prepared by larch and Chinese fir exhibited a large specific surface area, pore volume, and high degradation efficiency of formaldehyde solution. After 280 min of irradiation with an ultraviolet light source, the degradation rates of the formaldehyde solution were 19.91% and 18.85%.

## Introduction

In recent years, formaldehyde pollution of indoor air has been widely studied. The environmental protection and development of safety methods to deal with indoor formaldehyde and other decoration pollution has become a research hotspot^1–4^. The photocatalytic degradation of formaldehyde with TiO_2_ has the advantages of energy savings, environmental protection, cleanliness and nontoxicity. It can degrade indoor pollutants and bacteria effectively^5–7^.

The research and application of TiO_2_ in the field of environmental protection have continuously increased^6,8–11^. Improving the catalytic activity of TiO_2_ and preparing TiO_2_ with an improved structure have become the main research directions. TiO_2_ prepared by biological templates has important research and application value in terms of environmental protection and functional materials^12–15^. Using natural biomaterials as templates, the precursors of materials were impregnated into the templates by chemical or physical methods. Then, the templates were removed by calcination to obtain materials with natural biological morphology and structure^16^. The materials prepared by biological templates can effectively replicate the unique fine structure of organisms, optimize the structure and improve the performance.

Liu sintered TiO_2_ with good catalytic effect by using butterfly wings and chloroplasts as biological templates^17^. Luo prepared TiO_2_ materials with special properties and structures by using common filter paper as a template in the laboratory^18^. Li prepared TiO_2_ with a leaf network structure by the biological template method^19^. Chen et Al. synthesized TiO_2_ with a lignin template, and it exhibited a high photolytic activity^20^. Wood can also be used as a template to prepare certain oxides. The artificial Z-type reaction system of TiO_2_ was prepared by five different wood templates. It was found that the hydrogen production of TiO_2_ from wood templates was greatly improved by depositing precious metals^21^. Luo prepared TiO_2_–wood charcoal composites by using wood as a template, and it showed extraordinary adsorption ability for BPA^22^. Li et al. fabricated C/SiC-ZrC composite ceramics from pine and oak wood and improved their compression strength^23^.

As a natural biomass material, wood spices possess different microstructures. The preparation of a biomimetic TiO_2_ material using wood as a template can improve the photocatalytic degradation efficiency by inheriting and optimizing the structure of the wood template, which is helpful for indoor air environment control. In this study, poplar, paulownia, Chinese fir and larch were selected as templates to prepare TiO_2_. The effects of wood species on the photocatalytic degradation of formaldehyde and the microstructure of TiO_2_ were investigated. The visible light degradation of TiO_2_ based on wood templates was optimized by metal doping.

## Results and discussion

### Photocatalytic degradation of formaldehyde by TiO_2_

**Figure 1:**
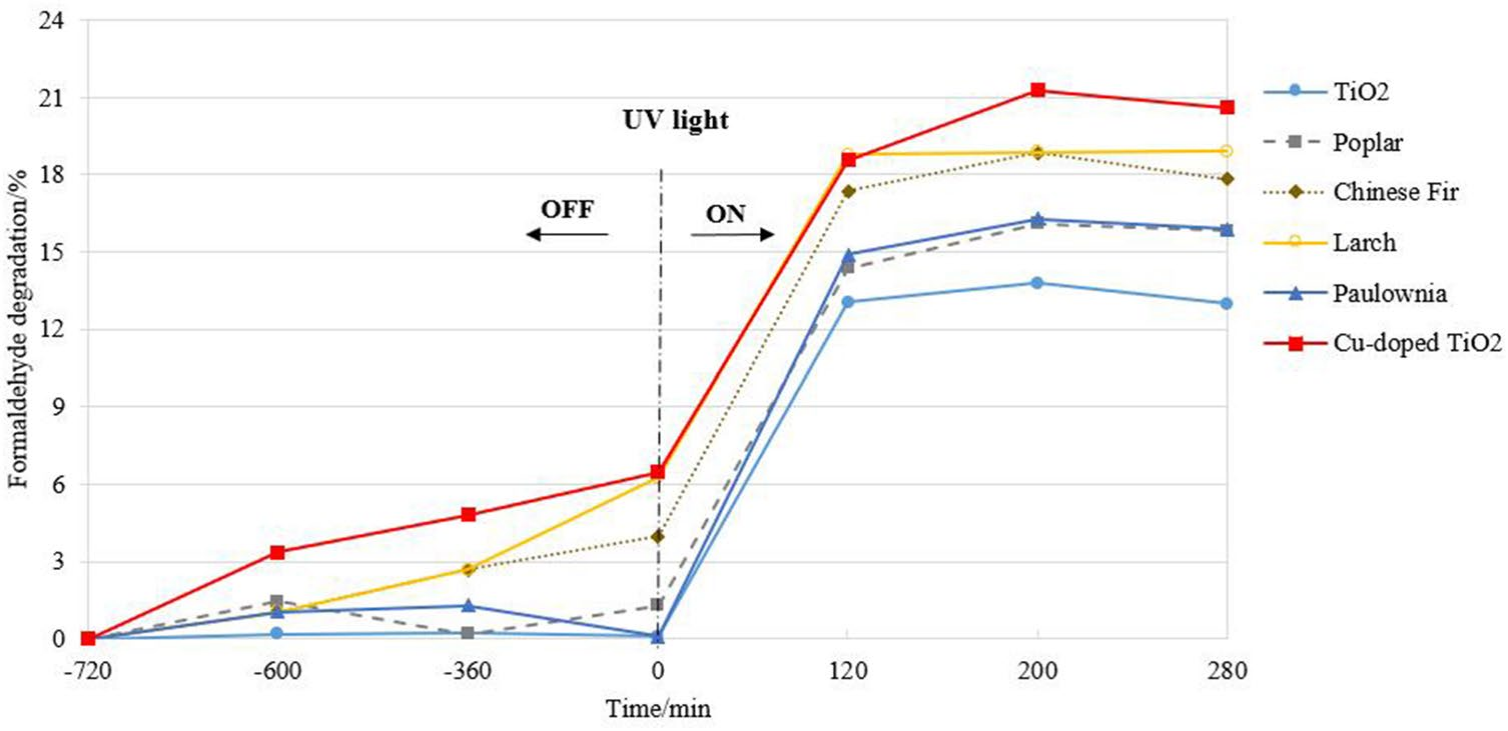
Degradation of the formaldehyde solution by different wood-templated TiO_2_ before and after UV light irritation.

Figure 1 shows the degradation of formaldehyde aqueous solution by TiO_2_ under different light illumination conditions. Before turning on the ultraviolet light source, it was determined whether, or not, the four kinds of wood template TiO_2_ need ultraviolet light to stimulate electrons to produce photodegradation. Therefore, the TiO_2_ adsorbed formaldehyde in solution first. Among the four types of wood templates, TiO_2_ prepared from larch and Chinese fir showed an improved adsorption of formaldehyde solution without ultraviolet (UV) light.

The effects of the four types of TiO_2_ prepared with poplar, Paulownia, Chinese fir and larch templates on the formaldehyde concentration showed no significant difference during the first 2 h. However, the concentration of formaldehyde solution treated with TiO_2_ prepared with larch and Chinese fir templates decreased after 6 h, and this trend continued to increase as the standing time increased. The concentration of the formaldehyde solution with larch and Chinese fir-templated TiO_2_ decreased by 6.27% and 3.97%, respectively, at 12 h. However, formaldehyde desorption occurred in the solution with poplar- and Paulownia-templated TiO_2_ during static processing, which made the concentration of formaldehyde solution fluctuate significantly.

**Figure 2:**
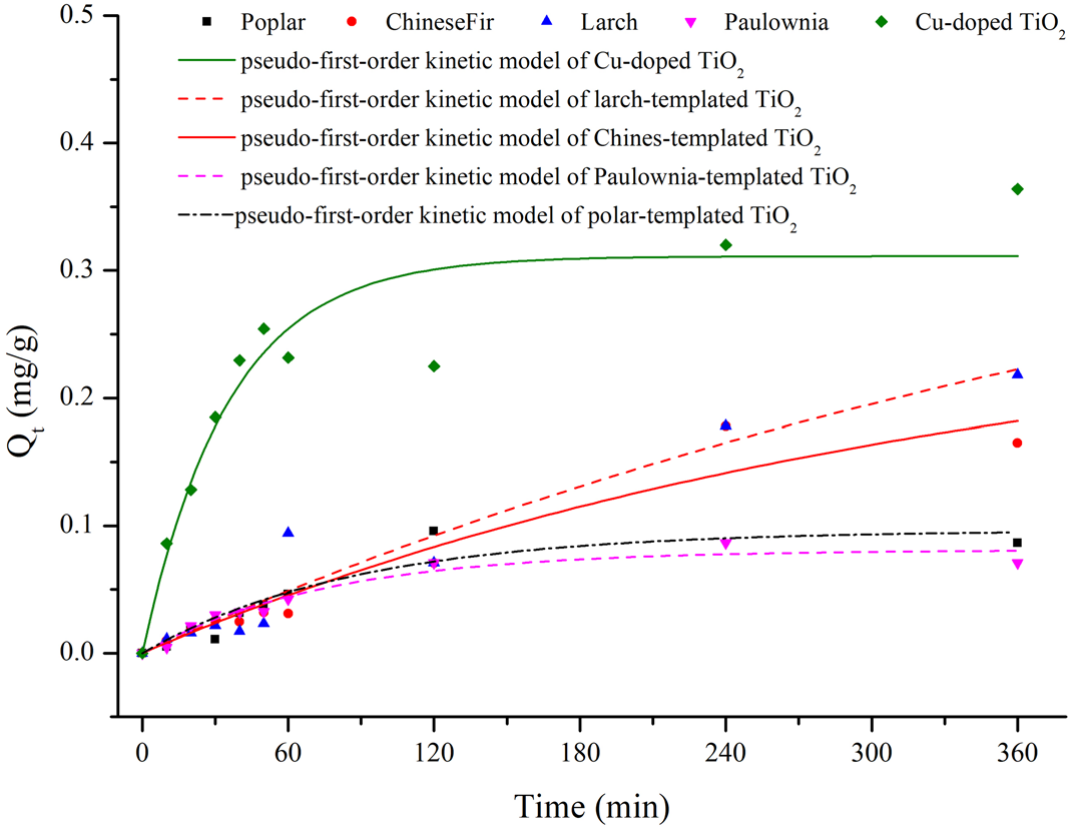
The adsorption kinetics curves of wood-templated TiO_2_ on formaldehyde.

To better understand adsorption phenomena, adsorption kinetics were also examined. As Fig. 2 and Table 1 showed, the adsorption followed pseudo-first-order kinetics, which is directly proportional to the dose. Cu-doped poplar-templated TiO_2_ showed the highest adsorption rate, followed by the hardwood-templated TiO_2_.

**Table 1:** Kinetic parameters obtained from the pseudo-first-order models of formaldehyde adsorption onto TiO_2_.

Type of TiO_2_	Qe,exp (mg g^−1^)	*k*_1_ (min^−1^)	Qe,cal (mg g^−1^)	*R*^2^
Poplar-templated TiO_2_	0.0865	0.0114	0.0964	0.88
Chinese fir-templated TiO_2_	0.1648	0.0030	0.2751	0.94
Larch-templated TiO_2_	0.2180	0.0020	0.4366	0.93
Paulownia-templated TiO_2_	0.0709	0.0133	0.0810	0.95
Cu-doped poplar-templated TiO_2_	0.3639	0.0283	0.3112	0.89

The catalytic degradation of formaldehyde in aqueous solution with copper-doped TiO_2_ occurred under visible light. Figure 1 shows that the concentration of formaldehyde solution treated with copper-doped poplar-templated TiO_2_ declined continuously within 12 h, and the highest degradation rate reached 6.48%. This was related to the increasing utilization of solar light by the copper-doped poplar-templated TiO_2_ and the broadening of the response wavelength of the TiO_2_. The doping of copper ions increased the utilization of solar light and broadened the response wavelength of the TiO_2_ prepared with the wood templates.

When the ultraviolet light turned on, four types of TiO_2_ showed an aldehyde-reducing effect on the formaldehyde aqueous solution. As shown in Fig. 1, the degradation of formaldehyde by the TiO_2_ prepared with the wood templates increased with an extension of the ultraviolet irradiation time, but the degradation efficiency was slightly different under the same illumination intensity. The concentration of formaldehyde solution treated with TiO_2_ prepared without a wood template decreased 13.05% after 2 h of ultraviolet light irritation. The maximum degradation efficiency of the formaldehyde solution was 13.8% within 280 min of irradiation. The degradation of the formaldehyde aqueous solution treated with wood-templated TiO_2_ under ultraviolet light was better than that of the formaldehyde solution without templated titanium dioxide. Copper-doped wood-templated TiO_2_ showed the highest degradation efficiency of 21.62%. The larch- and Chinese fir-templated TiO_2_ also exhibited improved degradation effects on the formaldehyde solution with degradation rates of 19.91% and 18.85%, respectively. To compare the UV degradation efficiency of all samples, the experimental data for the photocatalytic degradation of the formaldehyde aqueous solution by titanium dioxide with and without templates were integrated, and the reaction rate constant was calculated. The results showed that the apparent first-order rate constants for titanium dioxide without a template and poplar-templated TiO_2_, Chinese fir-templated TiO_2_, larch-templated TiO_2_, Paulownia-templated TiO_2_ and Cu-doped poplar-templated TiO_2_ were 0.0006, 0.0008, 0.0009, 0.0009, 0.0008 and 0.001, respectively. The TiO_2_ duplicated the microporous structure in the wood template and showed a stronger ultraviolet absorption ability. It absorbed an increased excitation energy to excite electrons, which is conducive to the photodegradation of titanium dioxide. The factors affecting the photocatalytic effect include the size, specific surface area and porosity of TiO_2_ grains. A smaller grain size and larger specific surface area increase the chance of contact between the particle surface and organic matter, which is conducive to the improvement of photocatalytic activity. This has been verified in the subsequent analysis of the microstructure characterization of the larch- and Chinese fir-templated TiO_2_.

### XRD

**Figure 3:**
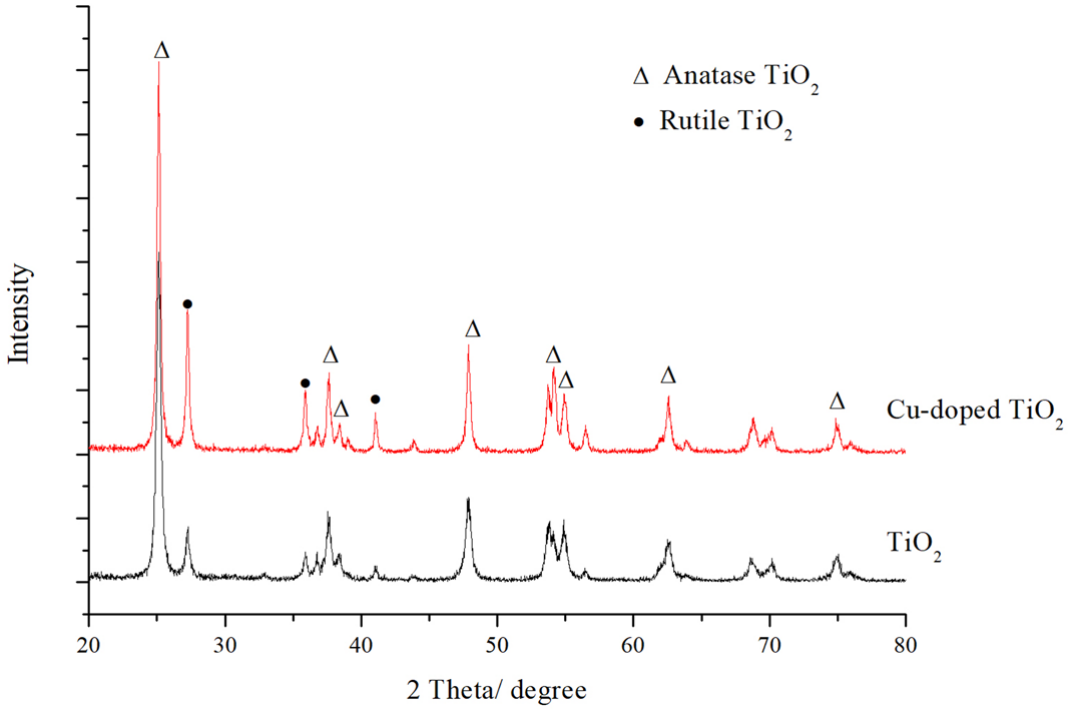
XRD analysis of TiO_2_ prepared with poplar template.

Figure 3 shows the X-ray diffraction (XRD) patterns of the poplar-templated TiO_2_ and copper-doped poplar-templated TiO_2_. Most of the TiO_2_ formed by calcination of the wood at 600 degrees comprised the anatase phase, and a minor amount comprised the rutile phase. According to the calculation, the average grain size of the poplar-templated TiO_2_ was 18.8 nm, and the crystallinity was 61.82%. The copper doping had no significant effect on the crystalline form of the TiO_2_ from the wood template, and its diffraction peaks were mainly anatase and rutile, which were the same as those from the TiO_2_ from the undoped wood template. After copper doping, the intensity of the main characteristic diffraction peaks from the TiO_2_ in the wood template increased as the crystallinity increased. The width of the peaks narrowed slightly, and the average grain size increased to 28.1 nm. The characteristic diffraction peak area of the TiO_2_ rutile-type copper-doped wood template obviously increased, and the content increased. It may be that the doping of copper reduced the phase transition temperature of the anatase, which increased the structure of the TiO_2_ rutile type of copper-doped wood template after the same calcination process. At the same time, there were no obvious characteristic peaks of copper-containing substances in the XRD patterns, which was related to the small amount of copper doping and the fact that copper doping does not form an independent phase but enters the crystal structure^24^.

### Raman spectroscopy analysis

**Figure 4:**
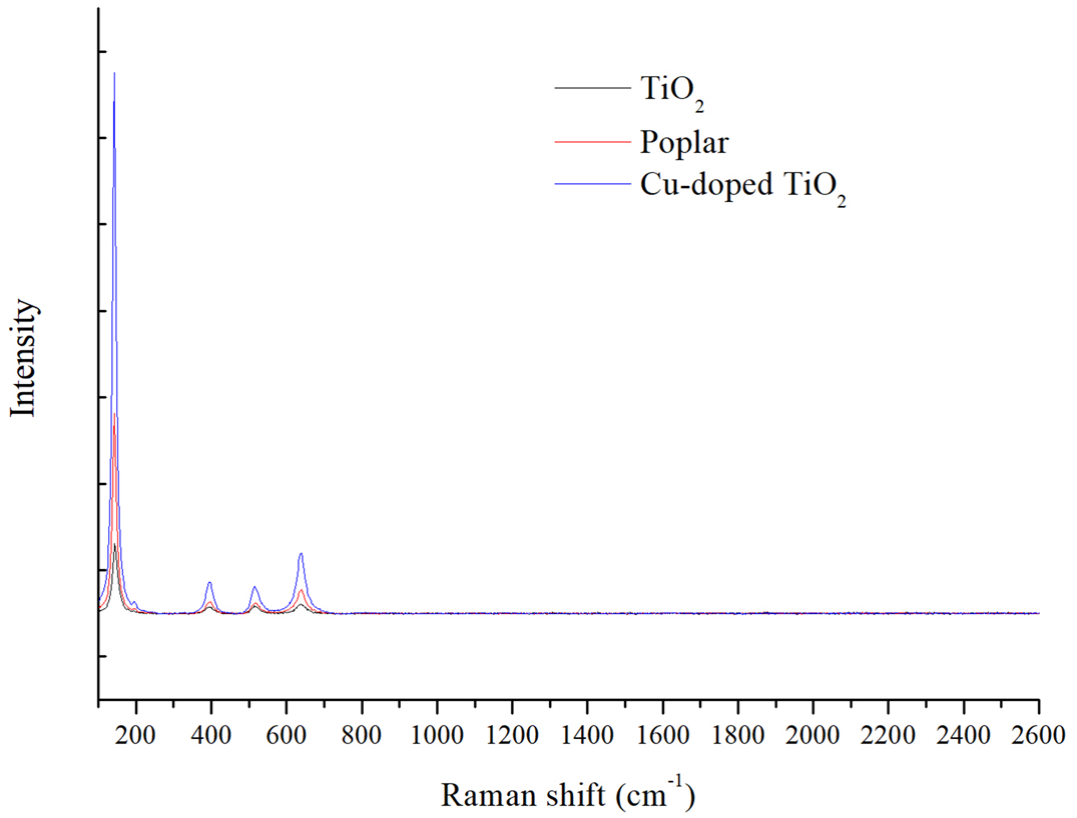
Raman spectra of TiO_2_.

Raman spectroscopy was used to study the internal structure of the catalyst. Figure 4 shows the Raman spectra of the TiO_2_ photocatalyst with and without a wood template and the Cu-doped wood-templated TiO_2_. In the figure, the peaks at 145 cm^−1^, 422 cm^−1^, 515 cm^−1^ and 639 cm^−1^ belong to the anatase phase of TiO_2_, while the peak at 216 cm^−1^ belongs to the rutile phase of TiO_2_. Because the lattice vibration of rutile is weaker than that of anatase, the other Raman characteristic peaks of rutile were covered by the anatase phase. It can be seen from the figure that the strongest anatase Raman peaks at approximately 145 cm^−1^ increased with the use of wood templates. The lattice vibration of the noble metal Cu was not measured for the same reasons diffraction peaks were not measured by XRD. The half width of the Raman peak at 145 cm^−1^ increased with increasing Cu doping. In addition, as the Cu loading of the noble metal increased, the position of the Raman peak of TiO_2_ redshifted, and the intensity of the peak gradually strengthened.

### SEM and TEM

**Figure 5:**
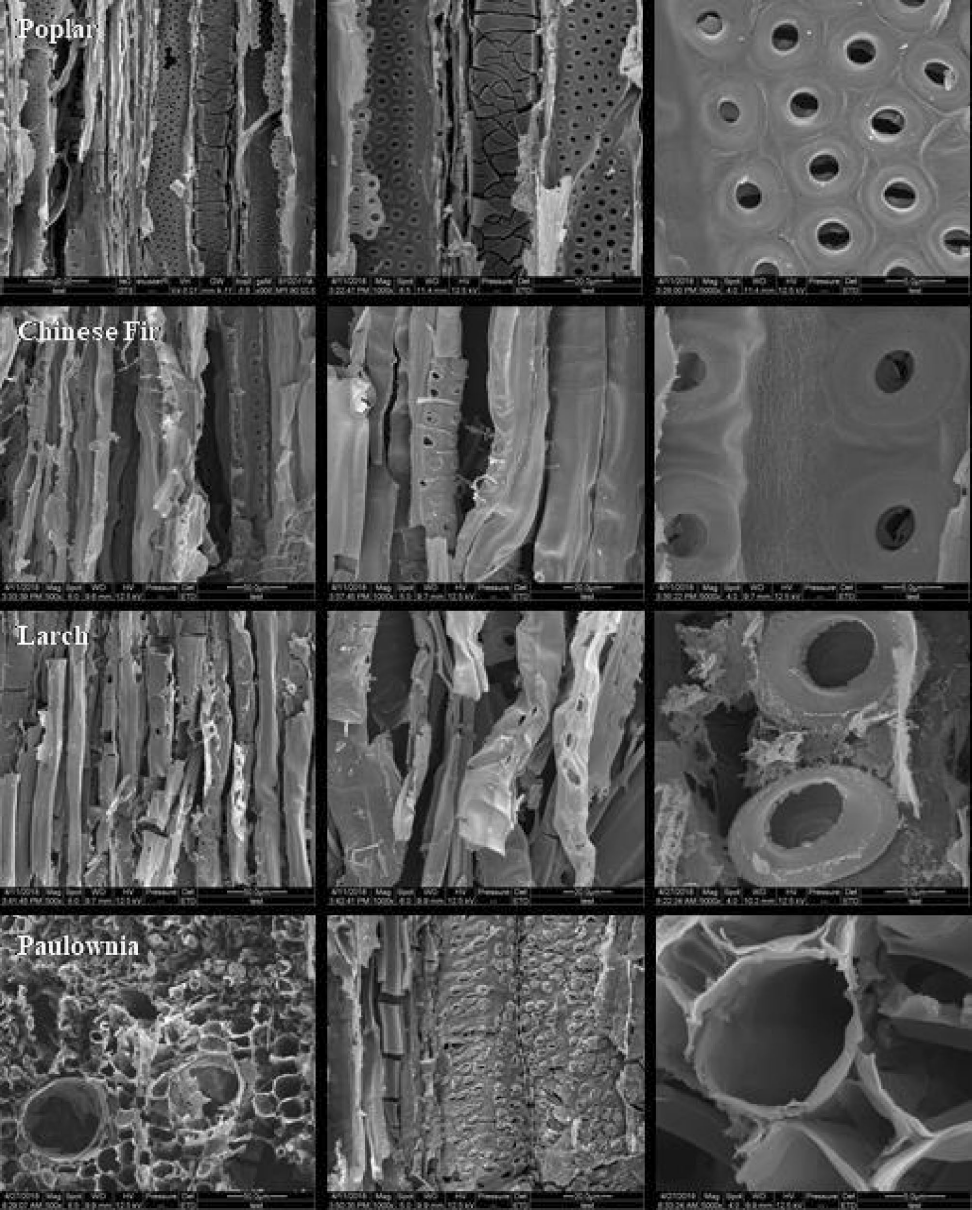
SEM images of wood-templated TiO_2_.

The microscopic morphology of wood-templated TiO_2_ was observed by scanning electron microscopy (SEM). The results are shown in Fig. 5. In the images of the Paulownia-templated TiO_2_, 50 µm macropores can be observed, which were mainly inherited from the Paulownia wood. At the same time, in the longitudinal section of the poplar, Chinese fir, larch and Paulownia, 10–20 µm cellular fibrous pores can also be observed, and the pore structure below 5 μm on the cell wall was duplicated. Pits are the main pathway for water transfer between cells in wood. In this study, the original pit plugs and pit membranes in the pits were partially removed through hydrothermal pretreatment, which enabled the TiO_2_ precursor to enter the pores of the wood cells and replicate the micropore structure of the wood effectively during sintering. During the precursor impregnation process, the precursor solution was evenly adsorbed or precipitated in the wood cell walls instead of accumulating in the pores in two ways: osmotic flow and diffusion. The transformation of the residual structure was achieved by optimizing the ratio of precursor solution and impregnation conditions. During the calcination stage, the precursor adsorbed on the cell wall was directly oxidized to produce the corresponding metal oxide phase, and at the same time, the original wood component was removed, and the porous structure of the wood was inherited from the oxide phase. The existence of a macropore structure is conducive to the improvement of photocatalytic performance, which is mainly because it provides a uniform and rapid diffusion channel for the adsorbed gas to the inside of the material, greatly increasing the reaction area with the measured gas.

**Figure 6:**
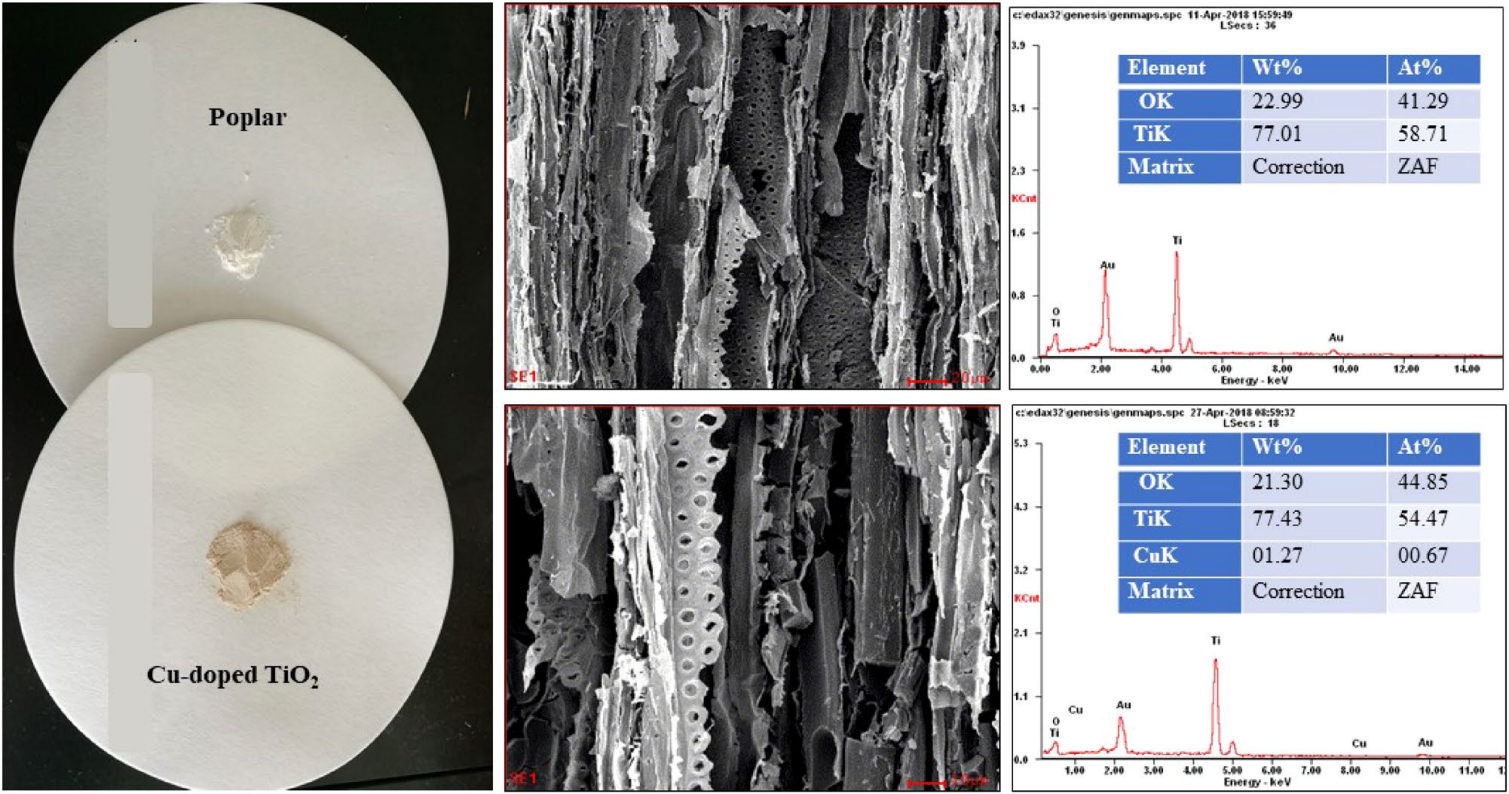
EDS analysis of the TiO_2_ prepared with the poplar template.

The color of the copper-doped wood-templated TiO_2_ was lavender red, while the undoped TiO_2_ in the wood template was a white powder, as shown in Fig. 6. The EDS results show that the preparation of wood-templated TiO_2_ with the method described in the experiment section removed the template material while retaining the microstructure of the wood template. Only Ti and O elements were detected in the sample. The copper doping process can indeed dope copper into the TiO_2_ of the wood template. However, the content was low mainly due to the small amount of doping. At the same time, the morphology of TiO_2_ doped with copper retained the fine structure of the wood. Copper, Ti and O together constituted the pore wall part of the product structure, while the original C in the wood structure was completely removed by high-temperature oxidation calcination.

**Figure 7:**
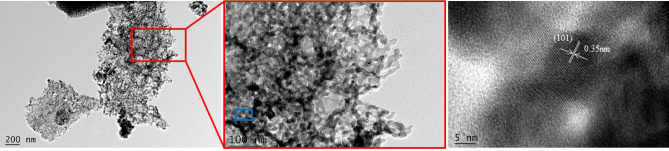
TEM analysis of TiO_2_ prepared with the poplar template.

In Fig. 7, the calcination products retained pit structure of wood. TiO_2_ particles were well crystallized and appear as crystalline hexagonal shape with sizes of 15–30 nm. A typical HRTEM image of the sphere wall as marked by a blue box was displayed in Fig. 7, in which a crystal lattice fringe with the spacing d value of 0.35 nm corresponds to the (101) crystal facets of TiO_2_ anatase type.

### Pore size and structure

**Figure 8:**
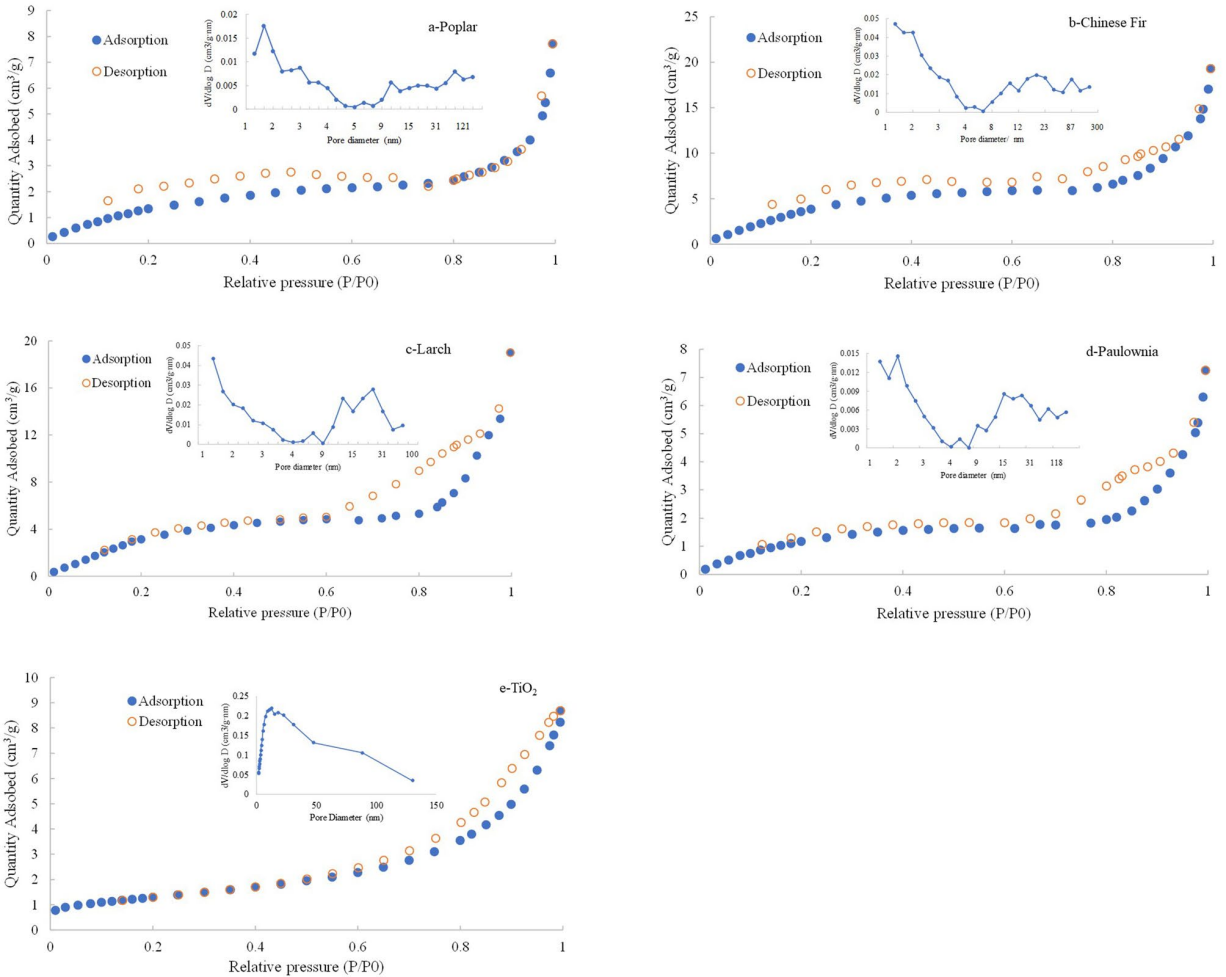
N_2_ adsorption/desorption isotherms of TiO_2_ prepared with different wood templates.

Figure 8 shows the nitrogen adsorption–desorption isotherms and pore size distribution of TiO_2_ synthesized with different types of wood templates. The nitrogen adsorption–desorption isotherm curve of the wood-templated TiO_2_ synthesized in this experiment is a typical type IV curve with an H3 hysteresis ring, which indicates that the synthesized TiO_2_ had a mesoporous structure. Moreover, there was a sharp jump at the relative pressure P/P = 0.8–1.0, which was caused by capillary condensation. At the same time, the ring cannot be closed at low pressure, which indicates that the material contained an irregular nanoporous structure. The results show that the pore size distribution of the wood-templated TiO_2_ was relatively wide.

The specific surface area, pore volume and pore size of the TiO_2_ were calculated with the adsorption isothermal data, as shown in Table 2. The specific surface area and pore volume of soft woods Chinese fir and larch were larger than those of hard woods Paulownia and poplar. The specific surface area of the Chinese fir was the largest, reaching 19.3991 m^2^/g. The TiO_2_ from the Paulownia template had the largest average pore size, followed by larch, poplar and Chinese fir. Different specific surface areas and pore sizes directly affected the microstructure of the wood-templated titanium dioxide.

**Table 2:** N_2_ adsorption measurement data for the wood-templated TiO_2_.

Samples	BET (m^2^/g)	Pore volume (cm^3^/g)	Adsorption average pore diameter (nm)	Desorption average pore diameter (nm)
Poplar	6.1109	0.0111	8.0548	12.8337
Chinese fir	19.3991	0.0272	6.8251	9.0057
Larch	17.5101	0.0283	8.9026	8.3464
Paulownia	5.3828	0.0107	9.2249	10.1131
TiO_2_ without a template	104.64	0.2530	9.9796	9.4455

At present, there are some reports on the synthesis of TiO_2_ using fiber templates to produce a porous material for photocatalysis degradation. In particular, the modification of TiO_2_ with nature plant fibers were found to improve the adsorption capacity, and it provide more efficiency for metal particles assembling on the nanopores. Different microstructures of TiO_2_ had a certain selectivity for the target degradation products. Compared with metallic oxide synthesized from other fiber template, such as rice husk, cotton fiber, tree leave, the size and shape of pore structure of wood templated TiO_2_ can be adjusted by choosing the appropriate wood species. An appropriate pore size was conducive to the transport and reaction of target degradation products into the active regions of the TiO_2_ in the wood template, which then produced different catalytic effects.

### UV–Vis spectroscopy

**Figure 9:**
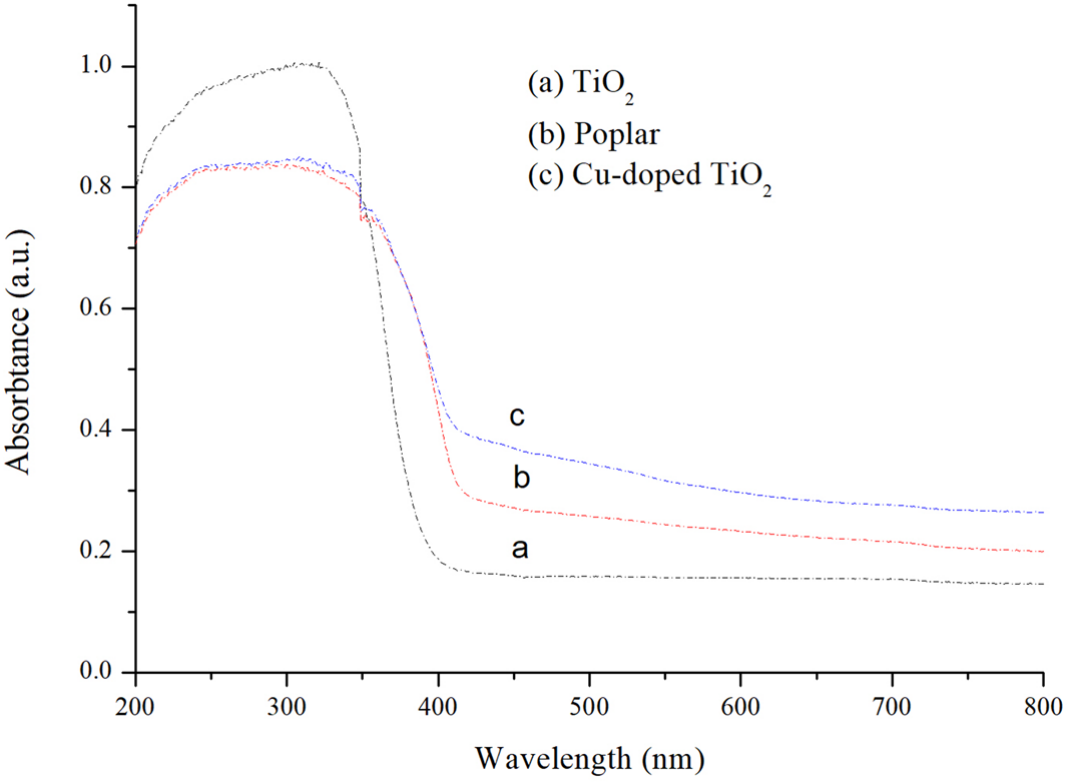
UV–Vis absorption spectra for TiO_2_.

**Figure 10:**
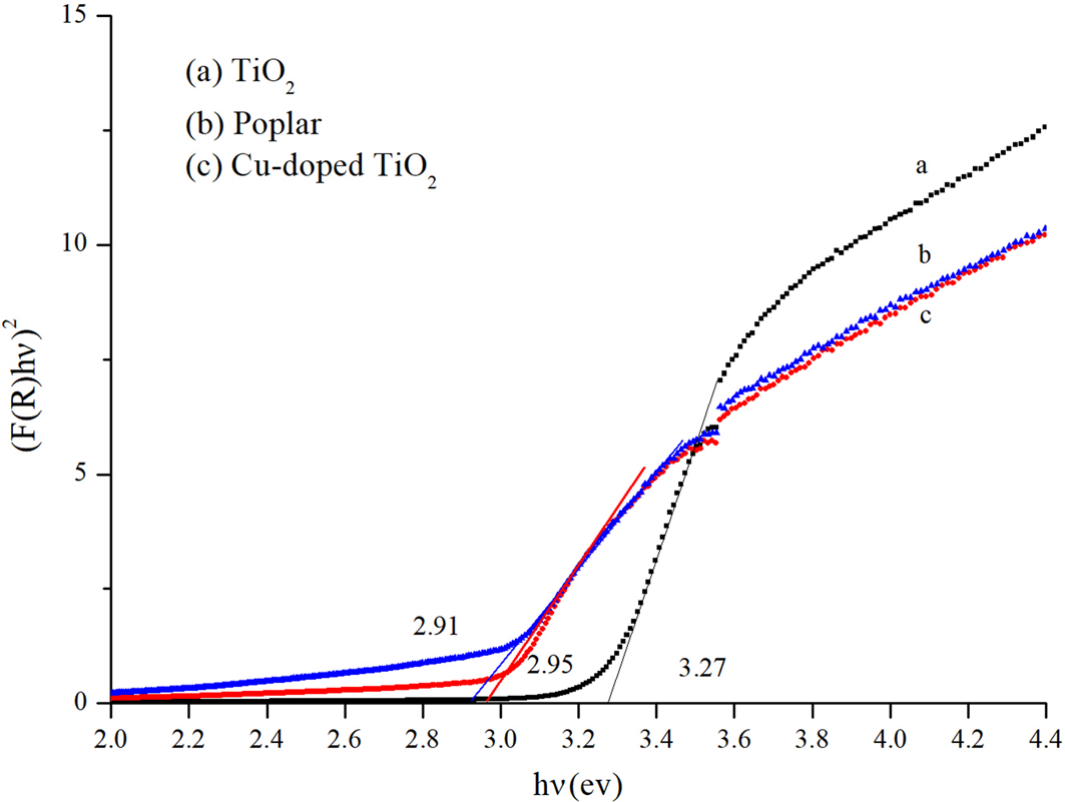
Plot of F(R)hν^2^ versus photon energy for TiO_2_.

Figures 9 and 10 show the UV–Vis DRS spectra for the different samples. Curve (a) is the UV–Vis spectrum of TiO_2_. It can be seen that the band gap width of the TiO_2_ was 3.2 eV, which demonstrated strong absorption in the UV region (in < 400 nm), but there was almost no absorption in the visible region. This indicated that light with a wavelength of 400 nm was not absorbed and utilized, so the energy contained in the photon in the visible region did not excite the electron transition from the valence band to the conduction band; (b) and (c) are the UV–Vis spectra of the wood-templated TiO_2._ It can be seen from the spectra that the absorption of ultraviolet light by the wood-templated titanium dioxide decreased, but the absorption in the visible light region increased. The wavelength of the absorption edge of the wood template redshifted, and the width of the band gap decreased.

## Conclusions

Wood treated by hydrothermal extraction was adopted for TiO_2_ preparation, and porous TiO_2_ with a wood-like microstructure was formed after high-temperature calcining. The TiO_2_ obtained by this method successfully reproduced the microporous and mesoporous structure of the wood. The wide pore size distribution ranged from 1 to 100 nm. The results show that the pore structure parameters of the TiO_2_ were related to the wood species. For the same preparation process, the specific surface area and pore volume of the TiO_2_ obtained from the larch and Chinese fir templates were higher than those obtained from the poplar and Paulownia templates. The crystal structure of the obtained TiO_2_ was a mixture of anatase phase and rutile phase. In the photocatalytic degradation testing of the formaldehyde aqueous solution, the TiO_2_ with a porous wood structure showed better absorption and utilization of light than the ordinary titanium dioxide. The formaldehyde degradation efficiency of copper-doped poplar-templated TiO_2_ reached 21.62% in 280 min.

## Methods

### Materials

The poplar (air-dry density 0.48 g/cm^3^), Paulownia (air-dry density 0.32 g/cm^3^), Chinese fir (air-dry density 0.47 g/cm^3^), and larch (air-dry density 0.40 g/cm^3^) were provided by Heilongjiang Yabuli Wood Industry Co., Ltd. The moisture content of the wood specimen was 4%. The butyl titanate, glacial acetic acid, acetylacetone and ammonium acetate were purchased from the Tianjin Guangfu Fine Chemical Research Institute.

### Preparation of TiO_2_ from the wood templates

To remove the extractives from the wood and open channels to transport the TiO_2_ precursor into the wood, hydrothermal treatment of the wood template was carried out. The poplar, Paulownia, Chinese fir and larch were sawed into small blocks with a size of 20 × 20 × 2 mm. Samples without nodules and decay defects were selected and treated in a hot water bath at 50 °C for 3 h. The samples were dried in a constant temperature drying oven at 60 °C for 6 h and then dehydrated with anhydrous ethanol. The precursor impregnating solution was prepared with butyl titanate, absolute ethanol, deionized water and glacial acetic acid in a molar ratio of 1:9:3:2. The butyl titanate was mixed with 2/3 volume of absolute ethanol to form liquid A, and 1/3 volume of absolute ethanol was mixed with deionized water and glacial acetic acid to form liquid B. The dewatered wood was impregnated in liquid A for 4 h, liquid B was dripped slowly into liquid A within 15 min, and then, the wood templates were taken out to air dry for 12 h under room conditions. Finally, the treated wood samples were put into a tubular furnace for high-temperature calcination. During the heating process, the temperature was slowly raised from room temperature to 260 °C and held for 40 min, then the temperature increased from 260 to 600 °C for 180 min.

### Preparation of copper-doped TiO_2_

To improve the visible light photocatalytic properties of the TiO_2_, the precursor solution was prepared with a molar ratio of butyl titanate: deionized water: absolute ethanol: glacial acetic acid: copper acetate = 1:3:9:2:0.0015. The specific operation methods were as follows: (1) butyl titanate was mixed with 2/3 of anhydrous ethanol to form liquid A; (2) the remaining 1/3 of anhydrous ethanol was mixed with glacial acetic acid to form liquid B; (3) copper acetate was dissolved in deionized water, stirred and dissolved to form liquid C; and (4) liquid C was added into liquid B, fully stirred and mixed to form liquid D. The wood treated by the hydrothermal pretreatment was put into liquid A. After being impregnated for 4 h under ultrasonic conditions, liquid D was slowly dripped into liquid A and reacted for 15 min. After the reaction, the wood samples were removed and air-dried for 12 h at room temperature. The copper-doped wood-templated TiO_2_ was prepared by calcination in a high-temperature tubular furnace.

### Photocatalytic degradation of the formaldehyde

In this study, a certain quantity of wood-templated TiO_2_ was put into the formaldehyde aqueous solution with a concentration of 10 mg/l. After mixing uniformly, the solution was irradiated by different light sources. The concentration of the formaldehyde solution after irritation was determined by spectrophotometry to investigate the TiO_2_ photocatalytic degradation of the formaldehyde solution.

### Characterization

The crystal structure of TiO_2_ from the wood template was characterized by XRD. Scanning electron microscopy (SEM) and EDS were used to observe the structure of the wood-templated TiO_2_. At the same time, nitrogen adsorption–desorption tests were carried out to measure the specific surface area, pore size and distribution of the samples. The pore structure replication of TiO_2_ from the wood template was also analyzed.
